# Breast biopsy in patients on anti-coagulants: is new guidance needed?

**DOI:** 10.1186/bcr3795

**Published:** 2015-11-05

**Authors:** Trupti Kulkarni, Andrew O'Connor

**Affiliations:** 1 University Hospital Aintree, Liverpool, UK; 2 Clatterbridge Breast Unit, Wirral, UK

## Introduction

Patients on anti-coagulation requiring breast biopsies are more at risk of bleeding. Newer anti-coagulants may not have a method for quantifying coagulation unlike the INR for warfarin. Also, some of these such as dabigatran do not have antidotes and rely on the body's ability to excrete the drug which may be altered by renal function. There are no up-to-date national guidelines on breast biopsy in such patients.

## Methods

A questionnaire for assessing practise of breast biopsy in patients on various anti-coagulants was devised. Responses were collected and analysed via Survey Monkey.

## Results

Seventy-eight complete responses were received and analysed. Thirty-eight per cent of respondents said they had local guidelines while 45 % used BSBR guidelines 2012. Sixty-three per cent would refer back to the GP/specialist in cases of warfarinised patients,14 % in cases of patients on clopidogrel and only 1 % of those on aspirin. Eighty-eight per cent of respondents did not have a policy for dabigatran and rivaroxaban. Practise was different in screening and symptomatic groups in 7 % due to the site of screening units away from A/E. Unit policy in warfarinised patients requiring vacuum-assisted biopsy (VAB) was not available to 38 %. Anecdotally, a number of radiologists reported that they would not perform VAB in patients on clopidogrel.

## Conclusion

There is a wide variation in practise while performing biopsies in patients on anti-coagulation including the newer anti-coagulants.

